# A Theoretical Study of 8-Chloro-9-Hydroxy-Aflatoxin B_1_, the Conversion Product of Aflatoxin B_1_ by Neutral Electrolyzed Water

**DOI:** 10.3390/toxins8070225

**Published:** 2016-07-21

**Authors:** René Escobedo-González, Abraham Méndez-Albores, Tania Villarreal-Barajas, Juan Manuel Aceves-Hernández, René Miranda-Ruvalcaba, Inés Nicolás-Vázquez

**Affiliations:** 1 UNAM–FESC. Campus 1. Chemical Sciences Department, Cuautitlan Izcalli, C. P. 54740, Mexico; renegerardo.escobedo@gmail.com (R.E.-G.); juanmanuel.is.acevesh@gmail.com (J.M.A.-H.); mirruv@yahoo.com.mx (R.M.-R.); nicovain@yahoo.com.mx (I.N.-V.); 2 UNAM–FESC. Campus 4. Multidisciplinary Research Unit L14 (Food, Mycotoxins and Mycotoxicosis), Cuautitlan Izcalli 54714, Mexico; 3 Esteripharma SA de CV. Atlacomulco 50450, Mexico; tvillarreal@esteripharma.com.mx

**Keywords:** Density Functional Theory (DFT), B3LYP, OSIRIS-Property-Explorer, neutral electrolyzed water, aflatoxin B_1_, 8-chloro-9-hydroxy-aflatoxin B_1_, quantum chemistry

## Abstract

Theoretical studies of 8-chloro-9-hydroxy-aflatoxin B_1_ (**2**) were carried out by Density Functional Theory (DFT). This molecule is the reaction product of the treatment of aflatoxin B_1_ (**1**) with hypochlorous acid, from neutral electrolyzed water. Determination of the structural, electronic and spectroscopic properties of the reaction product allowed its theoretical characterization. In order to elucidate the formation process of **2**, two reaction pathways were evaluated—the first one considering only ionic species (Cl^+^ and OH^−^) and the second one taking into account the entire hypochlorous acid molecule (HOCl). Both pathways were studied theoretically in gas and solution phases. In the first suggested pathway, the reaction involves the addition of chlorenium ion to **1** forming a non-classic carbocation assisted by anchimeric effect of the nearest aromatic system, and then a nucleophilic attack to the intermediate by the hydroxide ion. In the second studied pathway, as a first step, the attack of the double bond from the furanic moiety of **1** to the hypochlorous acid is considered, accomplishing the same non-classical carbocation, and again in the second step, a nucleophilic attack by the hydroxide ion. In order to validate both reaction pathways, the atomic charges, the highest occupied molecular orbital and the lowest unoccupied molecular orbital were obtained for both substrate and product. The corresponding data imply that the C_9_ atom is the more suitable site of the substrate to interact with the hydroxide ion. It was demonstrated by theoretical calculations that a *vicinal* and *anti* chlorohydrin is produced in the terminal furan ring. Data of the studied compound indicate an important reduction in the cytotoxic and genotoxic potential of the target molecule, as demonstrated previously by our research group using different in vitro assays.

## 1. Introduction

The contamination of food and feed with aflatoxins (a family of foodborne carcinogenic mycotoxins) is a serious problem for human and livestock health, and consequently has serious effects for agricultural economics. Once a commodity has been identified as being contaminated beyond a level fit for human or animal consumption, the problem arises as to what should be done with it. The producer of a highly aflatoxin-contaminated commodity may be faced with the problem of its disposal, unless some treatment may be found. Many experiments have therefore been performed to reduce the level of aflatoxins in contaminated materials using physical, biological or chemical approaches [1].

The value of the aflatoxins decontamination depends on the method used and the remaining cytotoxic and genotoxic potential of the treated material. The “*ideal*” decontamination process should have the following characteristics: easy to use, inexpensive, and free of the potential to produce compounds that remain or may reverse to form the parent mycotoxin, as in the case of nixtamalized products [2]. Recently, an environmentally friendly aflatoxin detoxification process was proposed in which maize grains were soaked in neutral electrolyzed water (NEW), filtered and dried at room temperature [3]. NEW treatment offers multiple advantages not found in other detoxification methods, including less chemical residues, more security, energy-savings and cost-effectiveness. NEW is produced by electrolysis of water—with no added chemicals, except for sodium chloride—in an electrolytic cell. In the anode, chloride ions and water molecules are transformed into chlorine oxidants such as hypochlorous acid (HOCl), hypochlorite ions (ClO^−^) and chlorine (Cl_2_) [4]. The antimicrobial mechanism of NEW depends mainly on three physicochemical properties: pH, oxidation-reduction potential (ORP) and available chlorine concentration (ACC) [5].

In a previous work, our research group proposed that the detoxification process eliminates the aflatoxin-associated cytotoxicity and genotoxicity effects due to the fact that NEW reacted with the double bond in the terminal furan ring of the aflatoxin B_1_ (**1**) molecule to yield 8-chloro-9-hydroxy-aflatoxin B_1_ (**2**) [3]. It was hypothesized that the most important factor in **1** transformation was the high level of ACC, taking into account that NEW contained primarily hypochlorous acid (≈95%), hypochlorite ions (≈5%) and trace amounts of Cl_2_. Although the analyses of **2** with Fourier transform ion cyclotron resonance mass spectrometry and ^1^H nuclear magnetic resonance have been reported [6], there is no specific information available concerning the structural, electronic and spectroscopic properties of the target molecule. Thus, the goal of this article is to inform novel and interesting results of **2** related to a theoretical study by applying quantum chemistry methods: (i) conformational analysis; (ii) the fully optimized molecule structure using Density Functional Theory (DFT) calculations at the level of theory defined by the Becke’s three-parameter hybrid functional (B3LYP), employing the 6-311++G(d,p) basis set; (iii) determination of the structural-geometrical parameters including bond lengths and angles; and (iv) calculation of electronic properties such as natural atomic charges, thermo chemistry, highest occupied molecular orbital (HOMO) and lowest unoccupied molecular orbital (LUMO), performed both in gas-phase and water as the solvent. Moreover, the molecular prediction about toxicological risks and some physicochemical properties of **2** were obtained using the OSIRIS-Property-Explorer (Actelion Pharmaceuticals Ltd, Allschwil, Switzerland). After a careful analysis of the obtained data, a plausible mechanism is now proposed in order to explain the formation of **2** in the process of aflatoxin detoxification with NEW.

## 2. Results and Discussion

**Figure 1:**
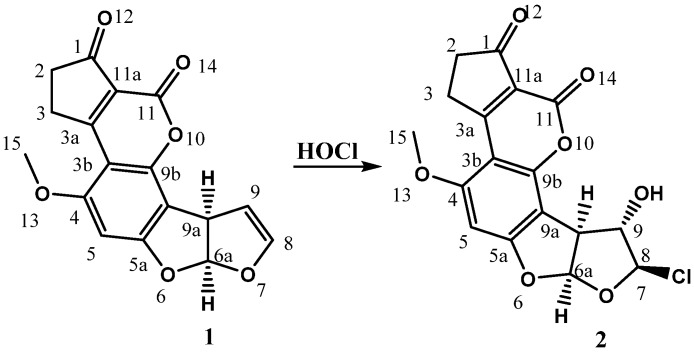
Formation of the 8-chloro-9-hydroxy aflatoxin B_1_ compound (**2**) from aflatoxin B_1_ (**1**) and hypochlorous acid from neutral electrolyzed water (NEW).

As it is well known, haloydrins are common chemical addition products of alkenes [7,8,9]. In the case of **1**, the reaction to generate the corresponding chlorohydrin was previously reported by Xiong et al. [6] and Jardon-Xicotencatl et al. [3], as a strategy for aflatoxin detoxification, specifying the achievement of 8-chloro-9-hydroxy-aflatoxin B_1_ stereoisomer (Figure 1).

### 2.1. Aflatoxin B_1_ Chlorohydrin Optimal Structure

**Figure 2:**
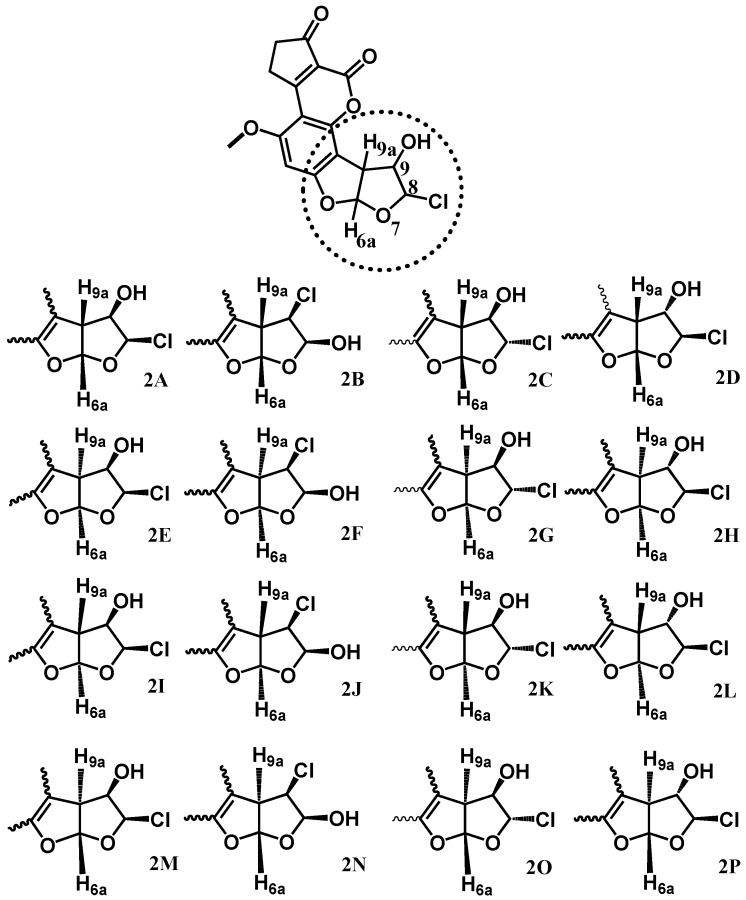
Sixteen structures optimized of the 8-chloro-9-hydroxy aflatoxin B_1_ compound (**2**) using the Density Functional Theory (DFT) level.

As stated above, sixteen possible isomers (stereoisomers and regioisomers) of **2** were fully optimized in the first phase of this study using the DFT level. The observed facts for these isomers were the chlorine and hydroxyl connectivity in position C_8_ or C_9_, and the conformational arrangement *syn* or *anti* regarding the hydrogen atoms H_9a_ and H_6a_, respectively (Figure 2).

The energy values of each isomer at the same level are summarized in Table 1. The less stable isomer corresponded to structure **2N** (–1,030,730.4 kcal/mol), as a consequence of the steric hindrance by the nearest atoms. In contrast, the most stable isomer was **2H** (–1,030,762.1 kcal/mol), with the chlorine and hydroxyl groups as expected according to this class of addition -*anti* position, with an energy difference of 31.77 kcal/mol. Thus, the structure **2H** was used in subsequent calculations and this isomer is named as **2**.

### 2.2. Reaction Mechanism

**Figure 3:**
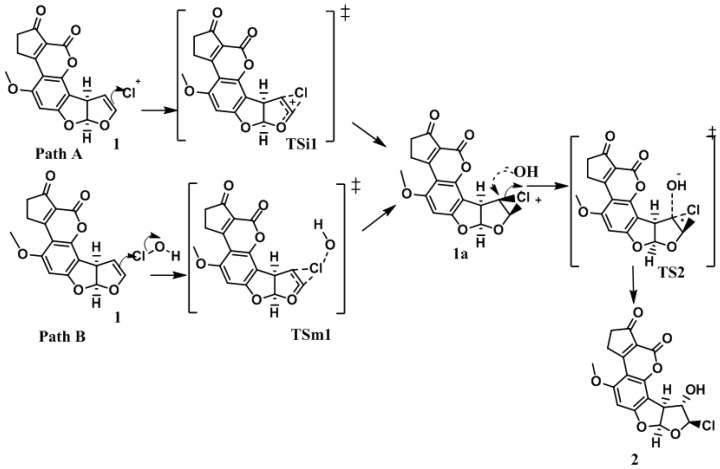
Initial pathways used in the determination of the theoretical reaction mechanism. **1**: Aflatoxin B_1_, **2**: 8-chloro-9-hydroxy-aflatoxin B_1_, **1a**: chloronium ion reactive intermediate, **TSi1**: ionic activated state 1 for path A, **TSm1**: molecular activated state 1 for path B, **TS2**: activated state 2.

In order to elucidate the formation process of **2**, two reaction pathways were considered according to the previously reported typical addition mechanism [10], one with only ionic species of the HOCl (Path A), and the second, considering the entire HOCl molecule (Path B). These two pathways are shown in Figure 3. Additionally, it is worth mentioning that both pathways were studied in gas and solution phases. 

The reaction begins—in path A and B—with an electrophilic attack by the double bond of **1** to the chlorenium ion (Path A: ionic), or hypochlorous acid (path B: molecular) succeeding the first ionic activated state (**TSi1** for path A) or the molecular activated state (**TSm1** for path B), followed by the production of the chloronium ion reactive intermediate (**1a**). In a second step, the hydroxide ion attained a nucleophilic attack to **1a**, which produced the second activated state (**TS2**), and concluded with the formation of **2** [11,12]. 

#### 2.2.1. Geometrical Parameters Analysis of the Structures Involved in the Mechanism

**Figure 4:**
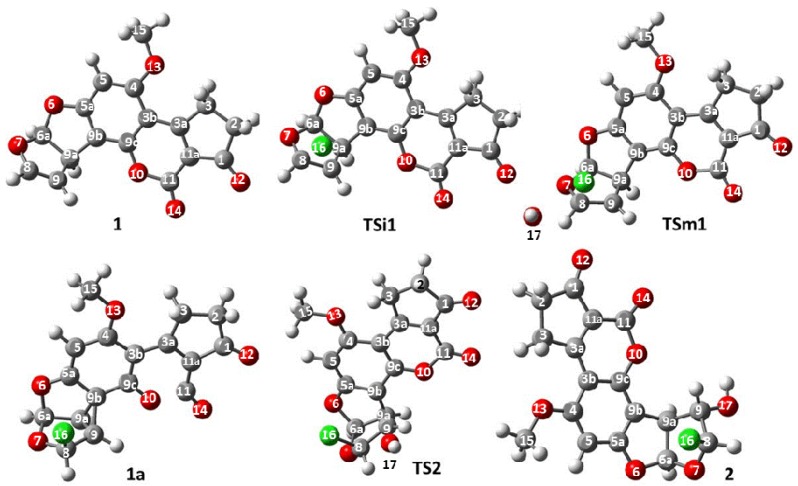
Optimized geometries for the reactive species of aflatoxin B_1_ (**1**), ionic activated state 1 in path A (**TSi1**), molecular activated state 1 in path B (**TSm1**), reactive intermediate (**1a**), activated state 2 (**TS2**) and 8-chloro-9-hydroxy-aflatoxin B_1_ (**2**).

Regarding the active species generation process, the optimized structures are shown in Figure 4. Selected geometrical parameters of the structures are summarized in Table 2 and Table 3. The reaction starts as suggested, with the interaction between the double bond at C_8_–C_9_ atoms of **1** and the chlorine atom (chlorenium ion or hypochlorous acid) to form the expected chloronium ion. The bond lengths (Table 2) of **TSi1** show an increase of the distance between C_8_–C_9_ atoms of **1**. Additionally, the distance of C_8_–C_9_ with the chlorine atom (1.95 and 2.35 Å, respectively) demonstrates a bond formation. However, a minor distance between C_8_–Cl atoms was observed, indicating a stronger interaction. Thus, a slight decrease in the distance and angle (Table 2) between C_9b_ and C_9_ atoms was also observed.

**Figure 5:**
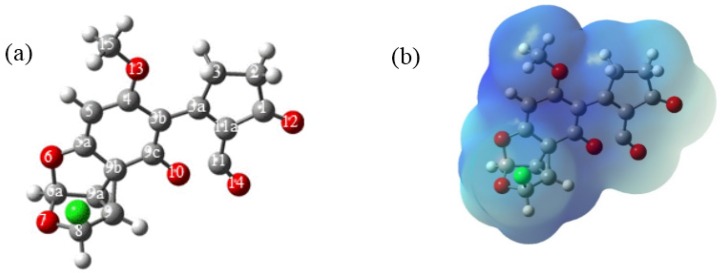
Carbocation intermediate: (**a**) structure of **1a** with the cyclopropane moiety formed among C_9_, C_9a_ and C_9b_. (**b**) electrostatic potential molecular surface of **1a**.

In the same way, a similar behavior in the activated state in path B was found. The bond at C_8_–C_9_ atoms increased in similar value to that of **TSi1**. On the other hand, the distance of C_8_–Cl atoms was shorter, while C_9_–Cl was longer compared to the same bonds in **TSi1**, indicating the preference of the chlorine addition at the C_8_ atom. The rest of the bonds in **TSi1** and **TSm1** agree with the experimental values and theoretical calculation for **1**. After the addition of the chlorine atom on the double bond, a reactive intermediate is produced. However, the proposed structure of the chloronium ion (Figure 3) was not obtained; instead, a non-classic carbocation was formed (Figure 5) [13].

The change in the structure of the reactive intermediate **1a** is a consequence of the interaction of the nearest aromatic system, which opens the chloronium ion and delocalizes the positive charge in the carbons of the benzene ring [13,14]. The electrostatic potential molecular surface (Figure 5b) showed a blue surface denoting the cationic character of **1a**; furthermore, the zones of the benzene and lactone rings had the most intense color confirming the electronic delocalization in these moieties. This process produced a cyclopropane moiety between C_9_, C_9a_ and C_9b_ with a value of 1.47 Å for C_9_–C_9a_ atoms, shorter than in **1**, and a value of 1.53 Å for C_9a_–C_9b_ atoms, which is longer in relation to the same bond in **1**. Finally, the bond length value for C_9_–C_9b_ atoms corresponds to 1.58 Å. Moreover, the angle for C_9_–C_9a_–C_9b_ decreases the value to 63.6°, which is consistent with the expected values for cyclopropane structures. 

The next reaction step is the nucleophilic attack of the hydroxide ion at C_9_ atom to form **2** via the second activated state (**TS2**). In this sense, the closeness of the hydroxide ion to C_9_ (2.51 Å) causes the enlargement of the C_9_–C_9b_ bond (from 1.582 in **1a** to 1.680 Å); additionally, the angle value at C_9_–C_9a_–C_9b_ also increases, signaling the breakage of the bond. The other bond angles and lengths values do not show significant changes. The attack of the hydroxide ion in the cyclopropane ring (instead of other sites with positive charge) is caused by the Bayer’s stress of the cycle and the high stability of the aromatic ring. Additional information presented in Table 2 and Table 3 are the geometrical properties of **2**. This molecule does not have experimental values of X-ray diffraction reported yet, though the theoretical values for **2** was compared with **1**, showing good congruity considering the structural differences. 

#### 2.2.2. Natural Charge in the Structures of the Reaction Mechanism

The natural populations of charges provide evidence of the electronic delocalization and the reactive site in the molecule. The charge analysis of the species involved in the reaction mechanism was also studied. The first step consisted of the interaction of the chlorine atom with the double bond to form either: **TSi1** or **TSm1,** causing a reduction of the positive charge on C_8_ atom. Meanwhile, C_9_ atom changed to positive charge as a consequence of partial bonds formed with the chlorine atom. The interaction between C_9b_ and C_9_ atoms was observed by a change in the distance and angle values in **TSi1** and **TSm1**, respectively. In addition, the natural charge analysis from **TSm1** (Table 4) showed that an increment in the negative charge on C_9b_ atom (−0.202) and the positive charge on C_9_ atom (0.108) was greater than that on C_8_ atom (0.097), assuming a possible interaction among them.

It is observed that when the C_9b_ atom forms a bond with the C_9_ atom, the electronic density in C_9c_ decreases. Thereby, O_10_ shares electronic density from unshared electrons, causing a drop in the negative charge of O_10_. The resonance effect delocalizes electrons, provoking an increment in the positive value at C_4_ (from 0.398 in **1** to 0.478 in **1a**). The electrodonating effect of the methoxy group is observed in decrement of the negative charge in O_13_ (from −0.539 to −0.479). Finally, C_5a_ also changes the value from 0.386 to 0.471. Considering the charge values, the atoms in the aromatic ring also change their values more closely to the **1**, recovering the original aromatic state.

#### 2.2.3. Thermochemistry of the Reaction

**Figure 6:**
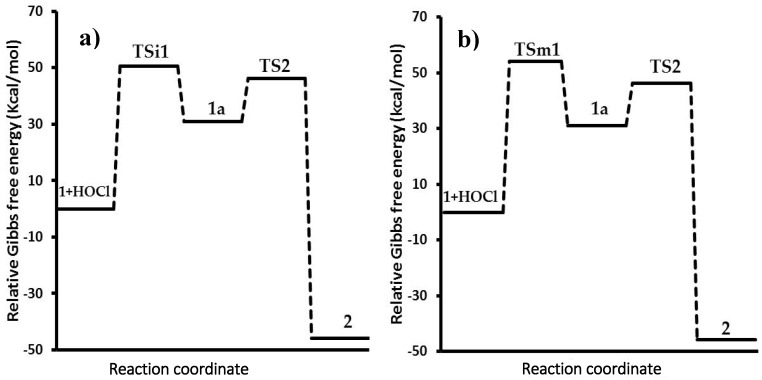
Energy profiles: (**a**) Path A: ionic; (**b**) Path B: molecular. **1**: aflatoxin B_1_, **2**: 8-chloro-9-hydroxy-aflatoxin B_1_, **1a**: reactive intermediate, **TSi1**: ionic activated state 1 for path A, **TSm1**: molecular activated state 1 for path B, **TS2**: activated state 2.

The relative Gibbs free energy profiles for these processes are plotted in Figure 6. The energy calculations for all species were made in aqueous solution; therefore, two profiles were constructed.

The path A (Figure 6, profile a) starts with the interaction of the double bond and the chlorine atom via activated state **TSi1** (Path A), which has an energy barrier in gas phase of 173.0 kcal/mol. However, considering the solvent effect, the energy barrier decreases to 50.4 kcal/mol, thus indicating a faster and feasible process. This change is a consequence of the stabilization of the ionic activated state (**TSi1**) by a polar solvent such as water [15,16,17], resulting in a faster reaction. The gas phase does not have this effect; consequently, an energy increment was observed. Since **1a** is an ionic reactive intermediate, the stability in water solution was greater (30.9 kcal/mol) than in the gas phase (150.0 kcal/mol) by the solvating effect. In the second step, the activated state (**TS2**) decreased the charge of the molecule (closely to the neutral charge) provoking a similar energy barrier in gas (62.2 kcal/mol) and solution (46.2 kcal/mol). Finally, the product is considered a polar molecule, which has a hydroxyl group. Thereby, the interaction of this group with water reduces the energy of the product up to −45.8 kcal/mol.

On the other hand, the thermochemical calculations from path B were performed in analogue form, considering the interaction between the double bond of **1** and the hypochlorous acid. In the calculations of gas and solution phases, both values were similar; the relative activation energy of the first activated state (**TSm1**) was 68.3 kcal/mol, while the energy barrier was 54.1 kcal/mol in solution. The less polarity of **TSm1** in comparison with **TSi1** allows more stability in gas phase and minor energy; however, partial charges formed were more stable in solution. Similar to path A, the reactive intermediate **1a** is produced and reacts with hydroxide ion to form **TS2**, with the same energy barriers mentioned previously. The results in gas phase showed a great difference in the activation energies (profiles not showed), in contrast with the solution process; for this reason, the process in gas phase is currently under study using other functional and basis sets.

#### 2.2.4. Frontier Orbitals Analysis

**Figure 7:**
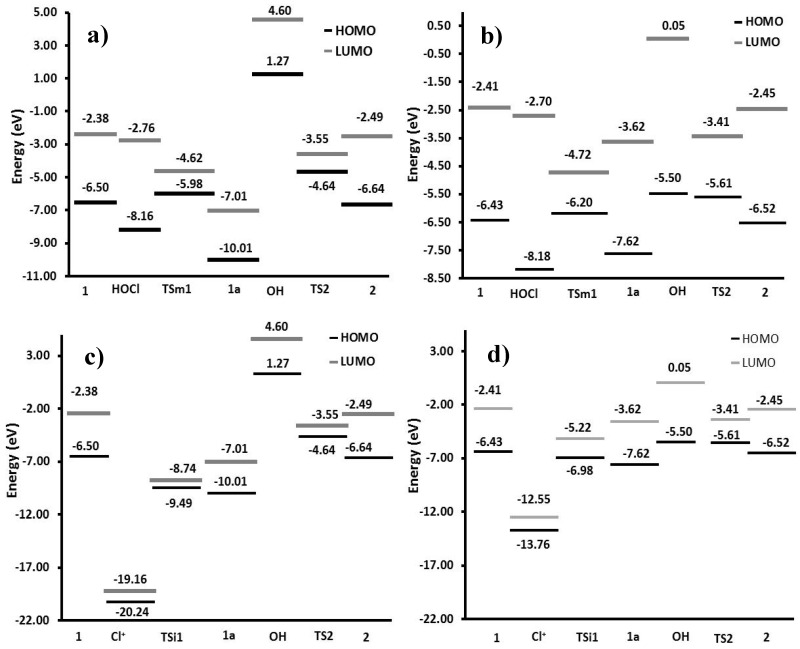
Energy gaps: (**a**) Path A in gas phase: ionic; (**b**) Path A in solution: ionic; (**c**) Path B in gas phase: molecular; (**d**) Path B in solution: molecular. **1**: aflatoxin B_1_, **2**: 8-chloro-9-hydroxy-aflatoxin B_1_, **1a**: reactive intermediate, **TSi1**: ionic activated state 1 for path A, **TSm1**: molecular activated state 1 for path B, **TS2**: activated state 2.

In order to describe the reactivity of the species involved in the reaction, a frontier molecular orbital analysis was made (Figure 7). HOMO–LUMO energy gaps of gas phase and solution were calculated and compared; the most feasible process has the smaller value. Thus, the interaction among the HOMO of **1** with the LUMO of HOCl or Cl^+^ in gas phase yielded energy gaps of 3.74 eV and −12.66 eV, respectively. In contrast, values of 3.73 eV and −6.12 eV were found in solution, which showed higher reactivity in aqueous medium. Although the interaction of **1** with the chlorenium ion has a minor value, this specie is unstable (gap value of 1.08 eV) and less probable to exist in the medium; thereby, the study was centered on path B.

**Figure 8:**
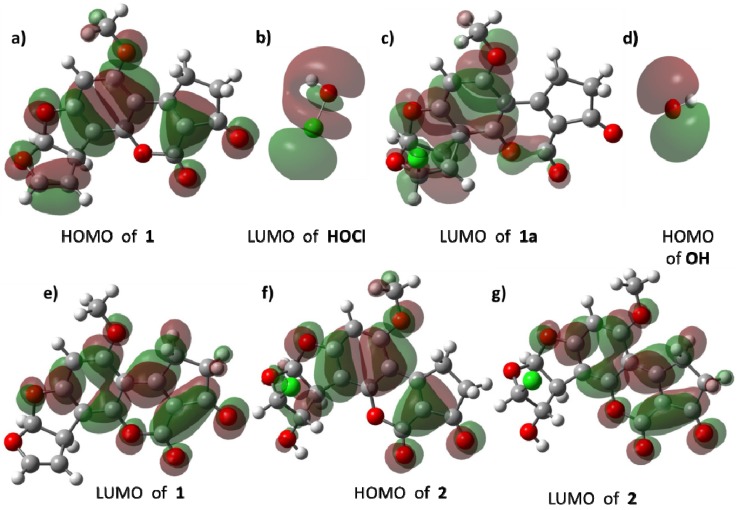
Frontier orbitals of the species involved in the reaction: (**a**) HOMO of **1**; (**b**) LUMO of HOCl; (**c**) LUMO of **1a**; (**d**) HOMO of **OH**; (**e**) LUMO of **1**; (**f**) HOMO of **2**; (**g**) LUMO of **2**. **1**: aflatoxin B_1_, **2**: 8-chloro-9-hydroxy-aflatoxin B_1_, **1a**: reactive intermediate.

Figure 8 shows the HOMO and LUMO of reactive species. In **1**, the HOMO (Figure 8a) is mainly distributed around the double bond of C_8_–C_9_ atoms and the aromatic system confirms the electrodonating effect of this moiety towards the LUMO of HOCl (Figure 8b). The LUMO of **1a** (Figure 8c) is concentrated in the aromatic ring, as a consequence of delocalization of the positive charge, and the cyclopropane motif reacts with the HOMO of the hydroxide ion (Figure 8d). Regarding product **2**, the distribution of its HOMO and LUMO is quite similar to the frontier orbitals of **1**.

#### 2.2.5. Bond Order and Reaction Mechanism

**Figure 9:**
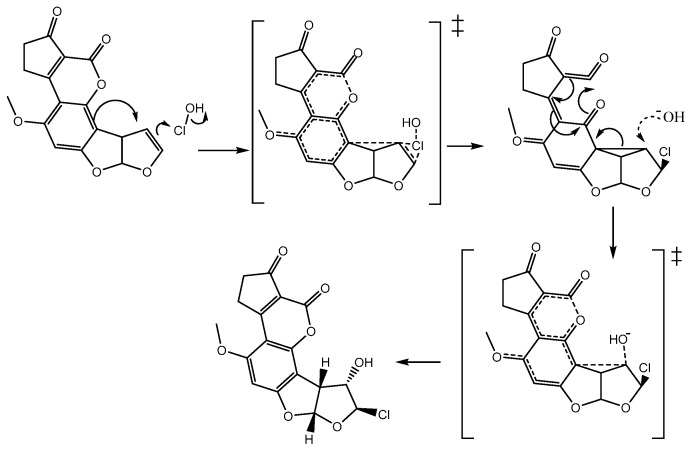
Reaction mechanism proposed with activated states and intermediates.

It is noteworthy to mention that, based on theoretical results, it is possible to propose the reaction mechanism with the activated states and intermediates (Figure 9). In summary, the double bond of **1** makes a nucleophilic attack on the chlorine atom of the hypochlorous acid breaking the Cl-OH bond; however, by assistance of the nearest aromatic system, the chloronium ion is not formed and a non-classic carbocation is obtained [13].

The bond order values of activated state **TSm1** were compared to that of the reactive **1** (Table 5). In **TSm1**, the C_8_–C_9_ bond had a bond order of 1.04 in contrast with **1,** which had a value of 1.869. In this same sense, the bond order among C_9b_ and C_9_ increased its value in **TSm1,** indicating a possible bonding interaction, coinciding with the decrease in the distance and bond angle between these atoms. On the other hand, the partial bond formed within C_8_–Cl and C_9_–Cl has values of 0.819 and 0.315, respectively, showing a stronger interaction among the C_8_ and Cl atoms. The bond orders in the aromatic ring carbon atoms were slightly smaller, and this fact is explained by the increment in bond length (Table 3).

The same analysis was made for **1a**, where it was possible to observe the bonding formation value between C_9b_–C_9_ (0.822), a decrement in the bond of C_11_–O_10_ and C_9b_–C_9c_, and an increment in the bond order among C_9c_–O_10_, when compared to **1**. All variations are in close agreement with the geometrical parameters formerly described. Finally, it is observed that bond order values of **2** are quite similar to those obtained for **1**, only with a significant decrement by the double bond break at C_8_–C_9_ atoms.

### 2.3. Spectroscopy Properties of ***1*** and ***2***

A complementary and interesting result was obtained from the calculation of Nuclear Magnetic Resonance (NMR) theoretical chemical shift for ^1^H, the coupling constant for three bonds (*J*^3^), and its correlation with those previously reported [6,18].

#### 2.3.1. Chemical Shift Prediction

**Figure 10:**
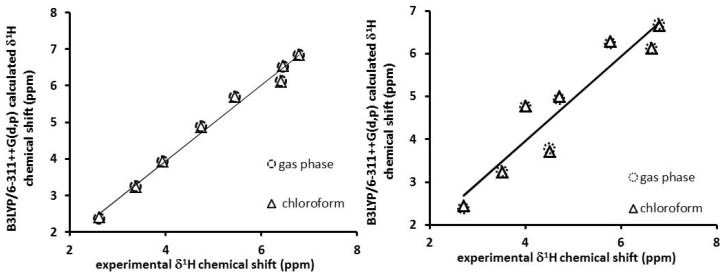
The linear regression between experimental and (Becke’s three parameter hybrid functional)B3LYP/6-311++G (d,p) calculated in solution and gas phase: (**a**) ^1^H NMR chemical shifts for **1**; (**b**) ^1^H NMR chemical shifts for **2**. **1**: aflatoxin B_1_, **2**: 8-chloro-9-hydroxy-aflatoxin B_1_.

The calculated values by the Gauge-Invariant Atomic Orbital method (GIAO) ^1^H NMR chemical shifts were plotted with and without the solvent effect versus the experimental data reported for **1** (Figure 10).

Linear regression analysis of the data set of ^1^H NMR shifts in gas phase provided the following results: a regression coefficient of 0.988 and a standard deviation of 0.193 ppm. The equation to describe the fit is:

∂T = 1.041∂*ex* − 0.2395 ppm (1)

where ∂T is a chemical shift predicted based on the experimental values; ∂*ex*, the slope, and the intercept is ppm, having a standard deviation of 0.0469 and 0.2432, respectively. The same analysis was made considering the solvent effect with a regression coefficient of 0.988 and standard deviation of 0.192 ppm, the equation to describe the fit is:

∂T = 1.034∂*ex* − 0.2077 ppm (2)

with a standard deviation of 0.0466 and 0.2421, respectively.

The calculated values in gas and solution phases of ^1^H chemical shift for **2** were also correlated with the experimental values. The regression analysis showed *r*^2^ coefficients of 0.882 in gas phase and 0.894 in solution; the standard deviations were 0.542 and 0.572 ppm, respectively. The equations to define these trends are:

∂T = 0.9875∂*ex* + 0.0088 ppm in gas phase (3)

∂T = 0.9933∂*ex* − 0.0291 ppm in solution (4)

The results of the calculated values for **1** and **2** were in close agreement with the experimental values.

#### 2.3.2. Theoretical Coupling Constant

The theoretical determination of the coupling constant (*J*^3^) was made for **1** and **2**. Comparing with the experimental values (Table 6), the predicted values are very close to the experimental values. In conclusion, both analyses reflected an adequate description of the experimental chemical shifts of ^1^H and *J*^3^ coupling constant by the selected method, theory level and basis set. 

### 2.4. Predicted Toxicological Properties for ***1*** and ***2***

The toxic, teratogenic and mutagenic effects of **1** have been amply studied [19,20,21]. However, these effects are a consequence of the epoxidation of the double bond at C_8_–C_9_ atoms and the covalent bonding of the epoxide to guanidine nucleotide in the DNA. The properties of **1**, **2** and the epoxide were predicted using the OSIRIS-Property-Explorer. The drug likeness may be defined as a complex balance of various molecular properties and structural features that determine whether a particular molecule is similar to the known drugs. The OSIRIS calculations for **1** and **2** and the epoxide are summarized in Table 7.

Results of the toxicity risk predictor showed that the compound with less risk of undesirable effects is **1**, which do not present risks of mutagenicity and tumorigenicity; however, this compound presented high irritating and reproductive effects. On the contrary, the epoxide presented medium risk for mutagenicity and tumorigenicity, though existing high risk to present irritating and reproductive effects. Finally, **2** does not present mutagenicity, which is in close agreement with previous reports [3,6], but the risk in other effects was high. The physicochemical properties of the compounds were also estimated. clog P, the logarithm of its partition coefficient between n-octanol and water log(c_octanol_/c_water_), is a property that describes the molecular hydrophobicity and varied from 1.56 to 4.95 (<5) [22]. In this research, the less bioavailable compound was **2**. As a consequence, the compound has poor permeability, which agrees with previous work [3,6], while the epoxide and **1**, had similar values. Drug solubility (expressed by log S) is an important factor to describe the absorption process. Poor solubility leads to poor absorption and biodisponibility [22]. The most soluble compound was **2**, indicating that this compound possesses the best absorption, movement in the blood stream, and better disposal by the urinary tract. The drug score (DS) is the combination of drug likeness, clog P, log S, molecular weight and toxicity risks in one handy value that may be used to judge the compound overall potential to qualify as a drug [22]. 

## 3. Materials and Methods

### 3.1. Optimization of the Structure Involved in the Mechanism

This study considered the AFB_1_ molecule’s maximum stability stereoisomer previously reported by our research group [23]. The connectivity of Cl^−^ and OH^−^ ions suggests sixteen possible stereoisomers for **2** due to the addition of chlorine and hydroxyl groups at C_8_ and C_9_ atoms. At a first stage, **2** was built with standard bond lengths and bond angles, using the PC Spartan 06 program [24]. Therefore, the first task was to establish the conformation of maximum stability. Thus, the geometry for each stereoisomer was fully optimized using DFT calculations, which were carried out using the Gaussian 09 program [25]. These calculations were carried out defined by the Becke’s three parameter hybrid functional (B3LYP) [26,27], which include a mixture of Hartree–Fock exchange with DFT exchange-correlation. The used basis set includes the split-valance and diffuse functions, 6-311++G(d,p) [28,29,30,31,32]. The default convergence criteria were also employed. The minima were verified performing a vibrational analysis. The structural parameters (bond lengths and bond angles) were analyzed at the same level, for intermediates and activated state (**TS**) of **1**, which were confirmed by frequency calculations. In all cases, intrinsic reaction coordinate (IRC) calculations were performed to test that the determined **TS**s connect with the proper reactants and products.

### 3.2. Atomic Charge Analysis

Natural Bond Orbital (NBO) was used for electron natural population analysis in the Gaussian program. Natural Population Analysis (NPA) was used for comparing differences rather than determining absolute atomic charges. This analysis was performed to investigate the electronic properties in the reaction mechanism [33]. The atomic charge was calculated at the same level of theory for all species involved in the reaction mechanism.

### 3.3. Thermochemical Parameters

Thermochemical values were estimated from frequency calculations, which included a thermochemical analysis of the system considering 298 K, 1 atm of pressure and the principal isotope for each element [34], all at the same theory level. The zero point correction to the electronic energy (ZPE) of the molecule was used to calculate the values of enthalpy and Gibbs free energy. The solvent effect was also calculated by using the self-consistent reaction field (SCRF) method and considering the Tomasi’s polarizable continuum model (PCM) at the same theory level, using water as a medium. For all energies, ZPE corrections were taken into consideration [35,36].

### 3.4. Frontier Orbital Analysis

The highest occupied molecular orbital-lowest unoccupied molecular orbital (HOMO–LUMO) gap is a typical quantity used to describe the dynamic stability of molecules [37]. The values of the orbital energy and the surface of the frontier orbitals were calculated using the same level of theory.

### 3.5. Bond Order

The natural bond orbital analysis provides an efficient method for studying intra and intermolecular bonding as well as its interaction. The calculation of bond order was developed to investigate the bond length before and after chemical transformation. The electron density of double and single bonds of the furan ring, clearly demonstrates a strong delocalization inside the molecule [33]. The calculation of bond order was developed using a natural bond orbital (NBO) analysis for all signaled species to investigate the electronic properties in the reaction mechanism [38,39,40].

### 3.6. Spectroscopy Properties

In addition, the ^1^H NMR chemical shifts were calculated using the Gauge Invariant Atomic Orbital method (GIAO method) taking into account the solvent effect and using tetramethylsilane as a reference. In the case of the coupling constant, the spin-spin option was employed at the same theory level [41,42].

### 3.7. Biological Properties

The toxicological risk and some physicochemical properties were obtained using the OSIRIS-Property-Explorer (Actelion Pharmaceuticals Ltd, Allschwil, Switzerland). The toxicological risk prediction process relies on a precompiled set of structural fragments that give rise to toxicity alerts in case they are encountered in the structure currently drawn. clog P and log S calculation method of OSIRIS is implemented as an increment system adding contributions of every atom based on its atom type. The drug likeness approach is based on a list of 5300 distinct substructure fragments with associated drug likeness scores. The drug likeness was calculated employing the score values of those fragments that are present in the molecule under investigation [43,44,45].

## Figures and Tables

**Table 1:** Conformational energy values of sixteen structures for the 8-chloro-9-hydroxy-aflatoxin B_1_ molecule (**2**).

Isomer	Energy (kcal/mol)	ΔE (kcal/mol)	Isomer	Energy (kcal/mol)	ΔE (kcal/mol)
2A	−1030759.6	2.5	2I	−1030734.1	28.0
2B	−1030759.6	2.5	2J	−1030730.4	31.8
2C	−1030761.9	0.2	2K	−1030735.9	26.2
2D	−1030759.1	3.0	2L	−1030735.3	26.9
2E	−1030759.6	2.5	2M	−1030734.1	28.0
2F	−1030759.6	2.5	2N	−1030730.4	31.8
2G	−1030759.1	3.0	2O	−1030735.3	26.9
2H	−1030762.1	0.0	2P	−1030735.9	26.2

**Table 2:** Calculated bond length for the reactive molecules.

Bond	Bond Length (Å)						
	1 Experimental	Theoretical					
		1	TSi1	TSm1	1a	TS2	2
C_3b_-C_4_	1.419	1.429	1.436	1.430	1.448	1.398	1.430
C_3b_-C_9c_	1.398	1.421	1.409	1.418	1.392	1.398	1.420
C_4_-O_13_	1.358	1.355	1.333	1.351	1.312	1.335	1.354
C_4_-C_5_	1.358	1.392	1.407	1.394	1.411	1.397	1.392
C_5_-C_5a_	1.401	1.393	1.380	1.391	1.369	1.380	1.393
C_5a_-O_6_	1.360	1.356	1.368	1.358	1.318	1.325	1.355
C_5a_-C_9b_	1.381	1.386	1.391	1.385	1.461	1.440	1.386
O_6_-C_6a_	1.440	1.450	1.413	1.441	1.483	1.495	1.355
C_6a_-O_7_	1.410	1.414	1.453	1.413	1.385	1.533	1.449
O_7_-C_8_	1.380	1.383	1.339	1.375	1.417	1.373	1.391
C_8_-C_9_	1.360	1.328	1.455	1.454	1.517	1.439	1.534
C_9_-C_9a_	1.500	1.516	1.473	1.489	1.470	1.544	1.551
C_9a_-C_6a_	1.550	1.562	1.563	1.559	1.549	1.467	1.555
C_9a_-C_9b_	1.500	1.508	1.513	1.511	1.529	1.517	1.500
C_9b_-C_9c_	1.387	1.384	1.393	1.384	1.464	1.437	1.383
C_9c_-O_10_	1.372	1.354	1.339	1.351	1.307	1.322	1.355
O_10_-C_11_	1.415	1.423	1.461	1.430	1.522	1.462	1.425
C_11_-O_14_	1.186	1.194	1.185	1.193	1.437	1.186	1.194
C_11_-C_11a_	1.455	1.449	1.447	1.448	1.175	1.444	1.448
O_13_-C_15_	1.430	1.427	1.441	1.429	1.452	1.437	1.428
C_1_-O_12_	1.193	1.209	1.203	1.208	1.200	1.206	1.208
C_8_-Cl	-	-	1.959	1.872	1.811	1.818	1.847
C_9_-OH	-	-	-	-	-	2.508	1.422
C_9_-Cl	-	-	2.354	2.427	-	-	-
Cl-OH	-	-	-	2.657	-	-	-
C_9_-C_9b_	-	2.560	2.410	2.530	1.582	1.680	2.592

**1**: Aflatoxin B_1_, **2**: 8-chloro-9-hydroxy-aflatoxin B_1_, **1a**: reactive intermediate, **TSi1**: ionic activated state 1 for path A, **TSm1**: molecular activated state 1 for path B, **TS2**: activated state 2.

**Table 3:** Bond angles (Å) of the reactive species.

Bond Angle	1 Experimental	Theoretical					
		1	TSi1	TSm1	1a	TS2	2
O_12_-C_1_-C_11a_	129.7	128.3	127.1	128.1	126.7	127.8	128.2
C_1_-C_11a_-C_3a_	112.7	111.2	111.1	111.2	111.3	111.2	111.2
C_1_-C_11a_-C_11_	124.8	125.7	124.8	125.5	124.2	125.3	125.6
C_3a_-C_11a_-C_11_	122.4	123.1	124.1	123.3	124.5	123.5	123.2
C_3_-C_3a_-C_3b_	128.0	127.7	128.0	127.8	128.1	128.0	127.8
C_11a_-C_3a_-C_3b_	121.2	121.1	120.7	121.0	120.9	120.8	121.1
C_11a_-C_11_-O_14_	128.8	129.8	131.7	130.2	134.6	131.8	130.0
C_11a_-C_11_-O_10_	116.5	113.7	112.6	113.5	110.9	112.3	113.6
O_14_-C_11_-O_10_	114.7	116.6	115.7	116.4	114.5	115.9	116.4
C_11_-O_10_-C_9c_	122.3	123.9	123.1	123.6	123.4	123.6	123.8
O_10_-C_9c_-C_9b_	115.0	116.8	116.6	116.8	115.3	116.2	116.7
O_10_-C_9c_-C_3b_	122.9	122.1	123.6	122.6	123.9	123.5	122.3
C_3b_-C_9c_-C_9b_	122.1	121.0	119.8	120.5	120.6	120.2	121.0
C_3a_-C_3b_-C_9c_	116.5	116.1	115.8	115.9	116.3	115.9	116
C_3a_-C_3b_-C_4_	125.8	126.1	126.1	126.1	125.7	126.0	126.2
C_4_-C_3b_-C_9c_	117.7	117.9	118.0	117.9	118.0	118.0	117.8
C_3b_-C_4_-O_13_	113.7	115.8	115.5	115.7	115.0	115.0	115.7
O_13_-C_4_-C_5_	123.4	122.7	122.5	122.7	122.0	122.4	122.7
C_3b_-C_4_-C_5_	123.0	121.5	122.0	121.6	122.9	122.6	121.6
C_4_-C_5_-C_5a_	115.5	117.3	117.0	117.3	118.0	117.8	117.3
C_5_-C_5a_-O_6_	122.4	123.0	124.3	123.3	124.2	124.1	123.4
C_5_-C_5a_-C_9b_	115.9	123.7	123.0	123.3	122.5	122.4	123.6
C_9b_–C_5a_–O_6_	111.8	113.3	112.7	113.4	112.9	113.2	113
C_5a_–C_9b_–C_9c_	115.9	118.6	120.2	119.3	116.6	117.3	118.7
C_9b_–C_9c_–C_9a_	134.1	132.2	131.3	132.1	129.0	131.3	131.8
C_5a_–C_9b_–C_9a_	109.8	109.1	108.4	108.6	105.6	106.1	109.4
O_6_–C_6a_–C_9a_	-	107.5	107.7	107.0	105.4	104.8	107.2
C_6a_–O_7_–C_8_	-	107.6	111.1	111.4	109.5	108.0	111.4
O_7_–C_8_–C_9_	-	115.0	109.9	108.5	105.3	104.7	107.1
C_8_–C_9_–C_9a_	–	108.6	109.2	109.5	108.6	106.1	102.7
C_9_–C_9a_–C_9b_	-	115.4	107.4	115.0	63.6	68.5	116.3
O_7_–C_8_–Cl	-	-	115.3	114.5	112.3	107.6	111.8
C_9_–C_8_–Cl	-	-	85.8	92.9	113.5	118.3	110.6
C_8_–C_9_–O_15_	-	-	-	-	-	73.9	106.3
O_15_–C_9_–C_9a_	-	-	-	-	-	180.0	111.8
C_l_–C_9_–C_9a_	-	-	116.0	117.4	-	-	-

**1**: Aflatoxin B_1_, **2**: 8-chloro-9-hydroxy-aflatoxin B_1_, **1a**: reactive intermediate, **TSi1**: ionic activated state 1 for path A, **TSm1**: molecular activated state 1 for path B, **TS2**: activated state 2.

**Table 4:** Natural charge distribution of the species involved in the mechanism.

Atom	Charge (e^−^)						
	1	HOCl	TSi1	TSm1	1a	TS2	2
C_4_	0.398	-	0.440	0.429	0.478	0.465	0.412
C_5_	−0.353	-	−0.334	−0.339	−0.371	−0.365	−0.353
C_5a_	0.386	-	0.378	0.392	0.471	0.441	0.396
O_6_	−0.535	-	−0.508	−0.533	−0.483	−0.508	−0.532
C_6a_	0.452	-	0.448	0.450	0.428	0.430	0.448
O_7_	−0.548	-	−0.203	−0.540	−0.537	−0.557	−0.561
C_8_	0.159	-	0.147	0.097	0.111	0.100	0.144
C_9_	−0.284	-	0.147	0.108	−0.179	−0.125	0.098
C_9a_	−0.272	-	−0.341	−0.341	−0.213	−0.257	−0.283
C_9b_	−0.183	-	−0.184	−0.202	−0.065	−0.096	−0.169
C_9c_	0.493	-	0.534	0.542	0.436	0.609	0.412
O_10_	−0.548	-	−0.570	−0.556	−0.537	−0.538	−0.546
C_11_	0.780	-	0.782	0.773	0.837	0.785	0.848
C_11a_	−0.291	-	−0.263	−0.195	−0.212	−0.312	−0.343
O_12_	−0.536	-	−0.496	−0.605	−0.488	−0.520	−0.557
O_13_	−0.539	-	−0.502	−0.528	−0.479	−0.516	−0.540
O_14_	−0.523	-	−0.474	−0.583	−0.462	−0.483	−0.543
C_15_	−0.207	-	−0.213	−0.213	−0.207	−0.208	−0.198
Cl	-	0.194	0.060	0.115	−0.014	−0.023	−0.096
O	-	−0.662	-	−1.119	−1.363	−1.184	−0.737
H	-	0.468	-	0.418	0.363	0.421	0.473

**1**: Aflatoxin B_1_, **2**: 8-chloro-9-hydroxy-aflatoxin B_1_, **1a**: reactive intermediate, **TSi1**: ionic activated state 1 for path A, **TSm1**: molecular activated state 1 for path B, **TS2**: activated state 2.

**Table 5:** Bond orders obtained for the reactions species.

Bond	Bond Orders					
	1	TSi1	TSm1	1a	TS2	2
C_3a_–C_11a_	1.509	1.549	1.517	1.551	1.528	1.512
C_11_–C_11a_	1.087	1.091	1.088	1.108	1.096	1.091
C_11_–O_14_	1.798	1.859	1.808	1.894	1.850	1.775
C_11_–O_10_	0.864	0.792	0.849	0.690	0.791	0.846
C_3a_–C_3b_	1.188	1.150	1.179	1.144	1.162	1.189
O_10_–C_9c_	1.025	1.051	1.020	1.187	1.113	1.031
C_3b_–C_9c_	1.247	1.293	1.256	1.380	1.348	1.260
C_3b_–C_4_	1.235	1.220	1.233	1.164	1.173	1.231
C_9c_–C_9b_	1.357	1.291	1.348	1.058	1.087	1.358
C_4_–O_13_	1.028	1.096	1.038	1.167	1.089	1.033
C_4_–C_5_	1.419	1.349	1.411	1.307	1.373	1.417
C_5_–C_5a_	1.322	1.388	1.331	1.456	1.383	1.324
C_5a_–C_9b_	1.337	1.294	1.332	1.050	1.110	1.330
C_9b_–C_9a_	0.988	0.949	0.971	0.870	0.875	1.001
C_9b_–C_9_	0.012	0.138	0.021	0.822	0.681	0.011
C_5a_–O_6_	1.029	0.997	1.024	1.145	1.123	1.030
O_6_–C_6a_	0.868	0.933	0.880	0.811	0.799	0.869
C_6a_–C_9a_	0.982	0.971	0.974	0.974	0.974	0.981
C_6a_–O_7_	0.930	0.859	0.926	0.980	1.004	0.927
O_7_–C_8_	0.967	1.084	0.991	0.921	0.877	0.959
C_8_–C_9_	1.869	1.097	1.104	1.002	0.997	0.983
Cl–C_8_	0.000	0.741	0.819	1.001	0.995	0.954
Cl–C_9_	0.000	0.376	0.315	0.022	0.025	0.022
Cl–O	-	-	0.215	-	0.012	0.014
O–H	-	-	0.832	-	0.819	0.764
C_9_–O_17_	-	-	0.472	-	0.118	0.936

**1**: Aflatoxin B_1_, **2**: 8-chloro-9-hydroxy-aflatoxin B_1_, **1a**: reactive intermediate, **TSi1**: ionic activated state 1 for path A, **TSm1**: molecular activated state 1 for path B, **TS2**: activated state 2.

**Table 6:** Coupling constant (*J*^3^) experimental and theoretical of **1** and **2**.

Coupling Constant (*J*^3^)				
Coupling	1		2	
	*J* Experimental (Hz)	*J* Theoretical (Hz)	*J* Experimental (Hz)	*J* Theoretical (Hz)
H_6a_–H_9a_	7.3	7.3	6.1	6.2
H_9_–H_9a_	2.5	2.5	-	-
H_9a_–H_8_	2.5	2.7	-	-
H_9_–H_8_	2.7	2.7	-	-
H_2_–H_3_	5.4	5.1	5.7	5.1

**1**: aflatoxin B_1_, **2**: 8-chloro-9-hydroxy-aflatoxin B_1_.

**Table 7:** OSIRIS-Property-Explorer (Actelion Pharmaceuticals Ltd, Allschwil, Switzerland) toxicological and physicochemical predicted properties.

	Property	1	Epoxide of 1	2
Toxicological risk	Mutagenicity	N	M	N
	Tumorigenicity	N	M	H
	Irritating effects	H	H	H
	Reproductive effects	H	H	H
Physicochemical properties	clog P	1.634	1.816	2.126
	log S	−3.266	−3.294	−3.182
	DL	−2.729	−2.017	−4.58
	DS	0.165	0.107	0.09

**1**: aflatoxin B_1_, **2**: 8-chloro-9-hydroxy-aflatoxin B_1_. **N** = no risk, **M** = medium risk, **H** = High risk. **DL** = drug likeness, **DS** = drug score.

## Abbreviations

The following abbreviations are used in this manuscript:

Å	Armstrong
ACC	Available chlorine concentration
AFB_1_	Aflatoxin B_1_
clog P	Logarithm of partition constant octane/water
Δex	Experimental chemical shift
DFT	Density Functional Theory
DL	Drug likeness
DS	Drug score
δT	Theoretical chemical shift
eV	Electron volt
GIAO	Gauge-invariant-atomic orbital
^1^HNMR	Hydrogen nuclear magnetic resonance
HOMO	Highest occupied molecular orbital
Hz	Hertz
IRC	Intrinsic reaction coordinates
*J*^3^	Three bond coupling constant
log S	Logarithm of solubility
log P	Logarithm of its partition coefficient between n-octanol and water
LUMO	Lowest unoccupied molecular orbital
NBO	Natural bond orbital
NPA	Natural population analysis
NEW	Neutral electrolyzed water
ORP	Oxidation-reduction potential
PCM	Polarizable continuum model
Ppm	Parts per million
SCRF	Self-consistent reaction field
TS	Transition or Activated state
ZPE	Zero point correction to the electronic energy
